# 

**Published:** 1872-07-01

**Authors:** 


Money Receipts to July 15th, 1872.—James F. Fitzsimons, $5.00; A. F. Chandler, $3.00; C. B. Wright, $1.50; J. P. Anthony, $2.00; R. E. McVey, $3.00; N. W. Abbott, $3.00; J. S. Kitchen, $3.00; A. N. Richardson, $1.50; W. T. Knapp, $3.00; T. O. Bannister, $3.00; J. W. Bond, $3.00; J. W. Harmon, $3.00; D. L. Manchester, $6.00.

